# Passive acoustic monitoring as a tool for early detection of invasive animal species: a systematic review and a case study

**DOI:** 10.1007/s10661-026-15873-2

**Published:** 2026-08-29

**Authors:** David Becker, Marit Kinga Kasten, Torben Weber, Ingo Grass, Thomas Hiller

**Affiliations:** 1 Ecology of Tropical Agricultural Systems, University of Hohenheim, Garbenstraße 13, 70599 Stuttgart, Germany; 2 Center for Biodiversity and Integrative Taxonomy (KomBioTa), University of Hohenheim, 70599 Stuttgart, Germany; 3 Institute of Landscape and Plant Ecology, University of Hohenheim, 70599 Stuttgart, Germany

**Keywords:** American bullfrog, Amphibian, Biological invasion, Exotic species, Nature conservation, Soundscape

## Abstract

Invasive animal species are spreading rapidly across the globe, creating an urgent need for efficient early-detection and monitoring tools. Passive acoustic monitoring has become an established method in biodiversity research, but its application to invasive species monitoring has been less systematically explored. Here, we combine a systematic literature review with a field-based case study to evaluate the potential of passive acoustic monitoring for invasive animal detection. We identified 26 studies on acoustic monitoring of invasive animals, mainly addressing amphibians (11 studies), birds and fish (five each) with most studies from the USA and Australia. The use of acoustic monitoring of invasive species has increased during the past decade, with recent studies applying automated detection, machine learning, and large-scale monitoring frameworks. As a case study, we further tested the feasibility of low-cost acoustic monitoring of the invasive American bullfrog (*Lithobates catesbeianus* or *Aquarana catesbeiana*) in southwestern Germany, combined with automated identification using BirdNET. We successfully confirmed bullfrog presence in five of the eleven monitored lakes, including sites close to protected nature reserves. Our results highlight the growing potential of passive acoustic monitoring of invasive species under field conditions. In combination with automated species detection, manual validation, and emerging real-time monitoring devices, passive acoustic monitoring becomes an increasingly powerful tool for early intervention and scalable management of biological invasions.

**Supplementary information:**

The online version contains supplementary material available at https://doi.org/10.1007/s10661-026-15873-2.

## Introduction

Globalization and worldwide trade have created unprecedented connectivity between regions and continents, allowing species to be transported far beyond their native ranges. This makes the introduction and establishment of non-native species a common consequence of human activity (Early et al., 2016; Hulme, 2009; Seebens et al., 2017). While many introduced species do not cause immediate or noticeable impacts, some can become invasive, resulting in substantial ecological and economic consequences (Pyšek et al., 2020; Simberloff et al., 2013). Invasive organisms can alter community structure, disrupt trophic interactions, facilitate pathogen transmission, and thereby contribute to the decline of native species (Bellard et al., 2016; Blackburn et al., 2019).

Once invasive populations establish and spread, eradication is often technically difficult and costly, making prevention and early monitoring the most cost-effective management strategies (Hulme, 2006; Leung et al., 2002; Lodge et al., 2016; Simberloff et al., 2013). However, monitoring rapidly spreading species, particularly across large spatial scales, is resource-intensive and often constrained by limited financial capacity for manpower. Traditional monitoring approaches such as transect walks or trapping include repeated site visits and can therefore be difficult to sustain over time, particularly when species are nocturnal, or only seasonally active (Haubrock et al., 2021).

In this context, novel monitoring technologies are increasingly explored to improve detection efficiency and spatial coverage. Acoustic monitoring has long been used to investigate animal behaviour, communication, and species occurrence, particularly for taxa that are more readily detected by sound than by visual observation. Early ecological applications were especially prominent in marine mammal research and, within terrestrial ecosystems, in studies of echolocating bats and vocal birds, where acoustic methods revolutionized the monitoring of otherwise cryptic and nocturnal species (e.g. Kalko & Schnitzler, 1989; Riede, 1993; Parsons & Szewczak, 2009). The development of affordable autonomous recording units increased digital storage capacity, and advances in automated signal processing transformed these approaches into passive acoustic monitoring (PAM), enabling standardized, continuous recording without the need for an observer to be present (Gibb et al., 2019; Shonfield & Bayne, 2017; Sugai et al., 2019). Together with advances in automated species recognition based on machine learning, PAM now enables efficient processing of large acoustic datasets and has become an increasingly important tool for biodiversity monitoring and ecological research (Kahl et al., 2021; Knight et al., 2017). By allowing autonomous, long-term monitoring across many locations simultaneously, PAM is particularly attractive for invasive species monitoring, where early detection, repeated monitoring, and broad spatial coverage are essential but often difficult to achieve using conventional survey methods.

Although PAM has become an established tool in biodiversity monitoring (Gibb et al., 2019; Shonfield & Bayne, 2017; Sugai et al., 2019), its application to invasive species monitoring has only recently gained attention (e.g. Ribeiro et al., 2022; Bota et al., 2024). Consequently, the available evidence is dispersed across different taxonomic groups, ecosystems, and monitoring objectives, making it difficult to evaluate the overall potential, limitations, and best practices of PAM for invasive species monitoring. To our knowledge, no comprehensive synthesis has evaluated how PAM has been applied across invasive species, the methodological challenges encountered, and the key considerations for its effective implementation.

Among invasive vertebrates, amphibians are particularly suitable candidates for passive acoustic monitoring and automated species identification due to their distinct mating calls (Adams & Pearl, 2007; Gerhardt, 1994; Urbina et al., 2020). The American bullfrog (*Lithobates catesbeianus* Shaw, 1802, nowadays also referred to as *Aquarana catesbeiana*, IUCN, 2025) is one of the most widespread invasive amphibian species worldwide and is posing a serious threat to native biodiversity (Adams & Pearl, 2007; Adriaens et al., 2013; Urbina et al., 2020). Native to eastern North America, American bullfrogs have established populations in South America, Europe, and Asia through aquaculture, pet trade, and intentional releases (Adriaens et al., 2013; Laufer & Waitzmann, 2002, 2020). Their high annual reproductive output, broad diet and tolerance of anthropogenic habitats facilitate rapid population growth and expansion (Adams & Pearl, 2007; Nussbaum et al., 1983; Urbina et al., 2020). In Europe, populations continue to spread locally, raising concerns for native amphibians and wetland ecosystems (Bota et al., 2024).

In southwestern Germany, American bullfrogs have established expanding populations in gravel pit lakes around the Upper Rhine region since the Early 2000s (Baden-Württemberg, near Karlsruhe) (Laufer & Waitzmann, 2002), where the nature conservation authorities are trying to limit the negative impacts of further regional spread (Laufer & Waitzmann, 2020; Wengerter et al., 2024). The proximity of several protected nature reserves to known bullfrog populations raises concern about the conservation of native species, particularly given the limited capacity for systematic monitoring. Therefore, automatically detecting newly colonized water bodies at an early stage is crucial to assess invasion dynamics and inform management responses. However, monitoring multiple lakes consistently over extended periods of time presents logistical and financial challenges for local authorities.

Given these challenges, this study combines a systematic literature review with a field-based case study to evaluate the potential of passive acoustic monitoring in invasive species monitoring. We synthesize existing applications across taxa to assess current developments, remaining gaps, and to derive key considerations for the application of PAM to invasive species monitoring. In parallel, we tested the feasibility of low-cost acoustic monitoring and automated species detection for invasive American bullfrogs in German lakes. Integrating evidence from the reviewed literature with a field-based case study allowed us to examine whether our tested methodology for automated acoustic species detection can provide reliable and management-relevant information that could support more efficient and scalable monitoring of invasive species.

## Passive acoustic monitoring of invasive animal species: systematic literature review

### Methods

To identify relevant literature, we used Scopus with the search string TITLE-ABS-KEY((bullfrog* OR "*Lithobates catesbeianus*" OR "invasive species" OR invasive) AND ("acoustic monitoring" OR "bioacoustic*" OR "passive acoustic monitoring") AND NOT "non-invasive") on 6.11.2025. This search resulted in 143 papers. We screened their abstracts to narrow down to 44 potentially relevant papers. After assessing the full texts of those 44 papers, we kept 26 relevant papers. We excluded studies that did not directly address the application of passive acoustic monitoring for invasive animal species, post-removal ecosystem restoration, invasive plants, or other monitoring methods such as telemetry.

### Results

**Fig. 1:**
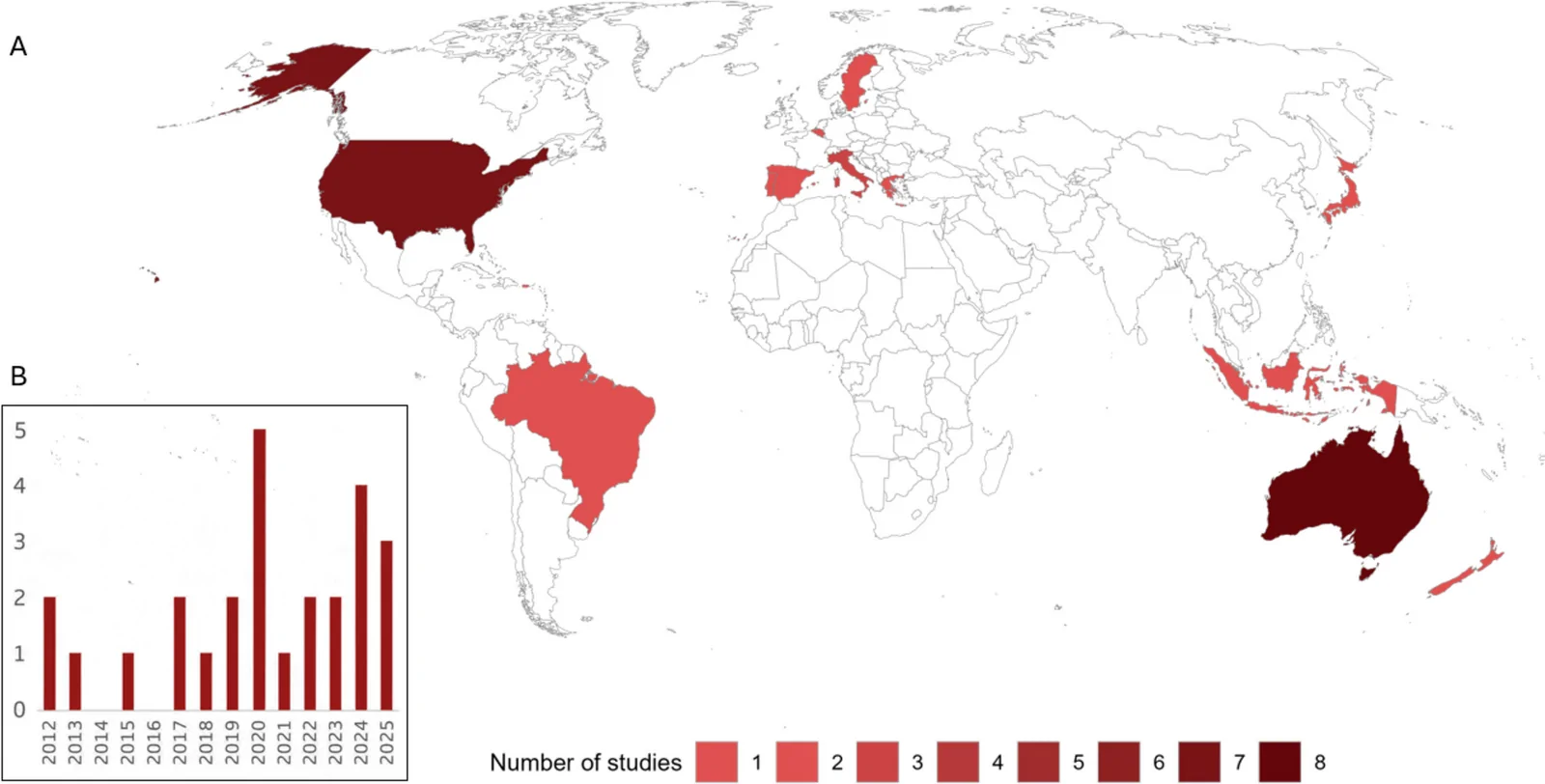
Global distribution and temporal development of studies using acoustic monitoring for the detection of invasive species. **A** World map showing the number of studies conducted per country. **B** Number of studies published per year between 2012 and 2025

The 26 identified papers covered a broad range of invasive animal taxa across terrestrial and aquatic ecosystems worldwide (Fig. 1A; Supplementary Table S1). Amphibians were the most frequently studied group (11 studies), followed by birds and fish (five each), arthropods (three), and mammals and reptiles (one each). Geographically, research was concentrated in a small number of regions, particularly Australia and the USA, while substantially fewer studies were conducted in Europe, Asia, and South America, and none were identified from Africa. This geographic pattern partly reflected repeated research on cane toads (*Rhinella marina*) in Australia and barred owls (*Strix varia*) in the USA (Fig. 1A). The earliest studies in our dataset were published in 2012, with an increasing number of publications in more recent years (Fig. 1B). Monitoring objectives ranged from early detection and range delimitation to occupancy assessment, long-term population monitoring, classifier development, and management support. Recording equipment was highly heterogeneous: Song Meter models were used in six studies, hydrophones or existing hydroacoustic recordings in seven, AudioMoths in three, and custom autonomous or real-time recording systems in four studies. Automated or semi-automated detection was applied in 17 studies, with BirdNET being the most frequently used individual classifier (four studies), followed by Raven Pro template detectors and PNW-Cnet models (two studies each). Nevertheless, manual or expert validation remained an important component and was explicitly reported in 18 studies; a further four studies used behavioural, experimental, laboratory, or methodological validation.

Most amphibian studies focused on invasive frogs and toads whose mating calls are species-specific, repetitive, and often produced in conspicuous choruses, facilitating automated detection. A substantial proportion of the literature addressed the invasive cane toad (*R. marina*), and most of them in Australia (Brodie et al., 2020; Kimura et al., 2025; Leung et al., 2025; Ribeiro et al., 2022; Roe et al., 2018; Taylor et al., 2017). Beyond technical advances in automated detection, cane toad studies have also addressed broader ecological and invasion-related questions such as long-term population dynamics and their effects on native biota. Long-term acoustic monitoring along the invasion front demonstrated that cane toads rapidly became a dominant component of local anuran communities, while most native frog species persisted, albeit with altered calling intensities (Taylor et al., 2017). At larger spatial scales, passive acoustic monitoring has been integrated with occupancy modelling to assess distribution patterns and habitat associations of invasive amphibians, including *R. marina*, revealing positive links between invasion success and anthropogenically modified environments (Ribeiro et al., 2022). More recent work has focused on early detection at invasion fronts, demonstrating that deep-learning models trained in established populations in Japan can reliably identify cane toad calls in areas where the species is rare or not yet established (Kimura et al., 2025). Similarly, continent-scale classifiers based on recordings from the Australian Acoustic Observatory were able to detect cane toads across diverse environments despite regional variation in call structure (Leung et al., 2025). The study by Leung et al. (2025) furthermore showed that the classifier was able to detect cane toads reliably even in regions where it had not been trained originally, suggesting that training models with large and geographically diverse datasets helps them remain robust to differences in background noise and regional variation in mating calls.

In Australia, beyond automated sensor networks, citizen-science approaches have expanded the spatial scale of acoustic monitoring. Rowley et al. (2019) introduced FrogID, a nationwide citizen-science program in Australia that collects smartphone recordings of calling frogs, which are subsequently validated by experts. Although the project targets Australian frog biodiversity more broadly, it has proven effective in detecting invasive amphibians, including species that have established populations outside their native range. By generating geo-referenced, expert-validated acoustic records at a continental scale, FrogID demonstrates how participatory passive acoustic monitoring can contribute to early detection and range tracking of invasive amphibians (Rowley et al., 2019). Even smartphone recordings are sufficient for reliable species identification in most cases, making large-scale acoustic monitoring feasible without dedicated recording hardware (Rowley et al., 2019).

Several additional studies addressed acoustic monitoring of other invasive anuran species, including the Cuban treefrog (*Osteopilus septentrionalis*), the American bullfrog (*L. catesbeianus*), and invasive marsh frogs (*Pelophylax* spp.) in Arizona, USA, and Europe (Bisconti et al., 2019; Bota et al., 2024; Huck et al., 2024; Tennessen et al., 2013). Tennessen et al. (2013) used passive acoustic monitoring to document the presence and chorusing activity of the invasive Cuban treefrog and to assess its effects on native treefrog calling behavior. Huck et al. (2024) used passive acoustic monitoring and occupancy modeling to assess anuran distributions in stream systems in Arizona, including the invasive American bullfrog, demonstrating how passive acoustic monitoring can quantify the occurrence of invasive amphibians in relation to habitat characteristics within mixed native–invasive assemblages. In Europe, passive acoustic monitoring has been applied to track the spread of invasive water frogs, particularly the American bullfrog (*L. catesbeianus*) and Balkan marsh frogs (*Pelophylax* spp.). Bota et al. (2024) used convolutional neural networks to reliably identify American bullfrog calls in invaded habitats, showing the potential of automated classifiers for long-term monitoring of invasive amphibians in temperate European landscapes. Similarly, Bisconti et al. (2019) used passive acoustic data to detect and assess the distribution of invasive marsh frogs in Europe, whereby species-specific call characteristics can be used to distinguish expanding invasive *Pelophylax* populations from native assemblages, even within this taxonomically complex frog group.

Compared with amphibians, reptiles make far less use of acoustic communication, which may explain why our literature survey yielded only a single study addressing the potential of acoustic monitoring for the Asian house gecko (*Hemidactylus frenatus*) (Hopkins et al., 2021). Originally restricted to Southeast Asia, this species has since spread across tropical and subtropical regions worldwide, where it is classified as invasive in several areas (Hopkins et al., 2021; Weterings & Vetter, 2018). Its characteristic “tik-tok” call, which gave the species its common name and results from its distinctive clucking vocalisations, is produced mainly by males during warmer months, with pronounced activity around sunset and roughly 30 min before sunrise (Hopkins et al., 2021). Building on this behaviour, the study by Hopkins et al. (2021) demonstrates how targeted acoustic monitoring could enable early detection of the species, helping to limit further spread.

Bird studies exclusively focused on the barred owl (*Strix varia*) in the USA, where its westward range expansion threatens the native and endangered spotted owl (*Strix occidentalis*). While barred owls use 2-phrased hoots, they can be easily distinguished from the spotted owls with their 4-note calls (Wood et al., 2020a, 2020b). Since 2020, two groups of scientists independently used different convolutional neural networks to detect the barred owls and developed models to monitor their distribution and population growth (Jenkins et al., 2025; Rugg et al., 2023; Wood et al., 2020a, 2020b). Lately, one group managed to employ real-time monitoring of the barred owl enabling them to target and lethally remove individuals (Wood et al., 2024).

An example of an invasive fish for which an automated classification method was developed and evaluated in Australia is the spotted tilapia (*Tilapia mariae*), which produces low frequency “knocks” or popping noises through muscular compression of its swim bladder (Kottege et al., 2012, 2015). More recently, the soniferous weakfish (*Cynoscion regalis*) became invasive on the Iberian Peninsula (since 2015) and is now threatening the native croaker (*Argyrosomus regius*) (Amorim et al., 2023). Among other studies, they developed approaches to discriminate invasive from local native fish inhabitants (Amorim et al., 2023; Demertzis et al., 2017; Raick et al., 2020). On top of that, Amorim et al. (2023) identified the potential for using this method of passive acoustic monitoring of this invasive species in the future. Pilot studies describing the sound characteristics of the red swamp crayfish (*Procambarus clarkii*) were conducted for potential future monitoring of this species (Buscaino et al., 2012; Hisyam et al., 2020).

Invasive mammals were highly underrepresented in our literature search. Lately, a dataset of 3500 vocalizations of the common brushtail possum (*Trichosurus vulpecula*) recorded in the field was presented by McEwen et al. (2024). Based on this dataset, McEwen et al. (2024) developed and evaluated a model capable of automatically classifying this invasive species in New Zealand.

Similarly underrepresented are studies on soil organisms. This gap is critical in contexts such as Arctic ecosystems, where invasive earthworms act as ecosystem engineers. Although direct acoustic detection of earthworms is difficult, their activity alters soil soundscapes, suggesting that indirect, large-scale monitoring may be possible through changes in belowground acoustic signatures (Keen et al., 2022).

Although most reviewed studies focused primarily on acoustic monitoring, a few combined PAM with complementary approaches. These included visual encounter and habitat surveys (Huck et al., 2024), as well as genetic analyses and interviews used to confirm the identity and distribution of invasive frogs (Bisconti et al., 2019). Other studies incorporated playback experiments, environmental measurements, or controlled mesocosm and laboratory experiments (Keen et al., 2022; Tennessen et al., 2013). In addition, eDNA metabarcoding and citizen science were proposed as promising complementary approaches for future invasive-species monitoring (Amorim et al., 2023).

The effective application of PAM for invasive species monitoring depends on the acoustic ecology of the target species, the invasion stage and monitoring objective, the spatial scale of monitoring, and the reliability of acoustic detection and classification. Based on the reviewed literature, Table 1 therefore addresses three central questions: Can the target species be monitored acoustically, how should the monitoring programme be designed, and how can the reliability of detections be ensured? It also highlights situations in which additional validation or complementary monitoring approaches may be required.

**Table 1:** Key considerations for the application of passive acoustic monitoring (PAM) to invasive species, synthesized from the reviewed literature

Key consideration	Implications for PAM design and application	Representative examples from the reviewed literature
Acoustic suitability and complementary methods	PAM is most suitable for species producing distinctive, sufficiently frequent, and temporally predictable sounds. Recorder placement and timing should reflect diel, seasonal, and habitat-specific activity. For weakly vocal, seasonally silent, or non-vocal species, PAM should be targeted or combined with complementary approaches	Invasive amphibians, barred owls, Asian house geckos, and soniferous fishes produce detectable signals suitable for PAM (Amorim et al., 2023; Bota et al., 2024; Hopkins et al., 2021; Wood et al., 2020a, 2020b). Earthworms and crayfish illustrate indirect or exploratory acoustic applications (Buscaino et al., 2012; Hisyam et al., 2020; Keen et al., 2022)
Monitoring objective, invasion stage, and spatial coverage	Monitoring design should reflect whether the objective is early detection, range delimitation, population monitoring, or management evaluation. Early detection requires high sensitivity and frequent processing, whereas established populations can be monitored using longer-term or spatially extensive recorder networks. Citizen science may further increase geographic coverage	PAM has supported early detection of cane toads, occupancy assessments of invasive amphibians, long-term population monitoring, nationwide citizen science, and management of barred owls (Jenkins et al., 2025; Kimura et al., 2025; Ribeiro et al., 2022; Rowley et al., 2019; Taylor et al., 2017; Wood et al., 2024)
Automated detection, classifier reliability, and validation	Automated classifiers facilitate analysis of large datasets but require representative training data and validation under relevant environmental and geographic conditions. Background noise, regional call variation, and acoustically similar native species may reduce transferability. Validation effort should increase when detections indicate new invasions or trigger management actions	Automated classifiers have been developed for cane toads, bullfrogs, fishes, barred owls, and brushtail possums. Manual or expert validation remains common, particularly for citizen-science records, invasion-front detections, and management-relevant alerts (Bota et al., 2024; Leung et al., 2025; McEwen et al., 2024; Rowley et al., 2019; Wood et al., 2024)

## Passive monitoring of the invasive American bullfrog: case study

### Methods

**Fig. 2:**
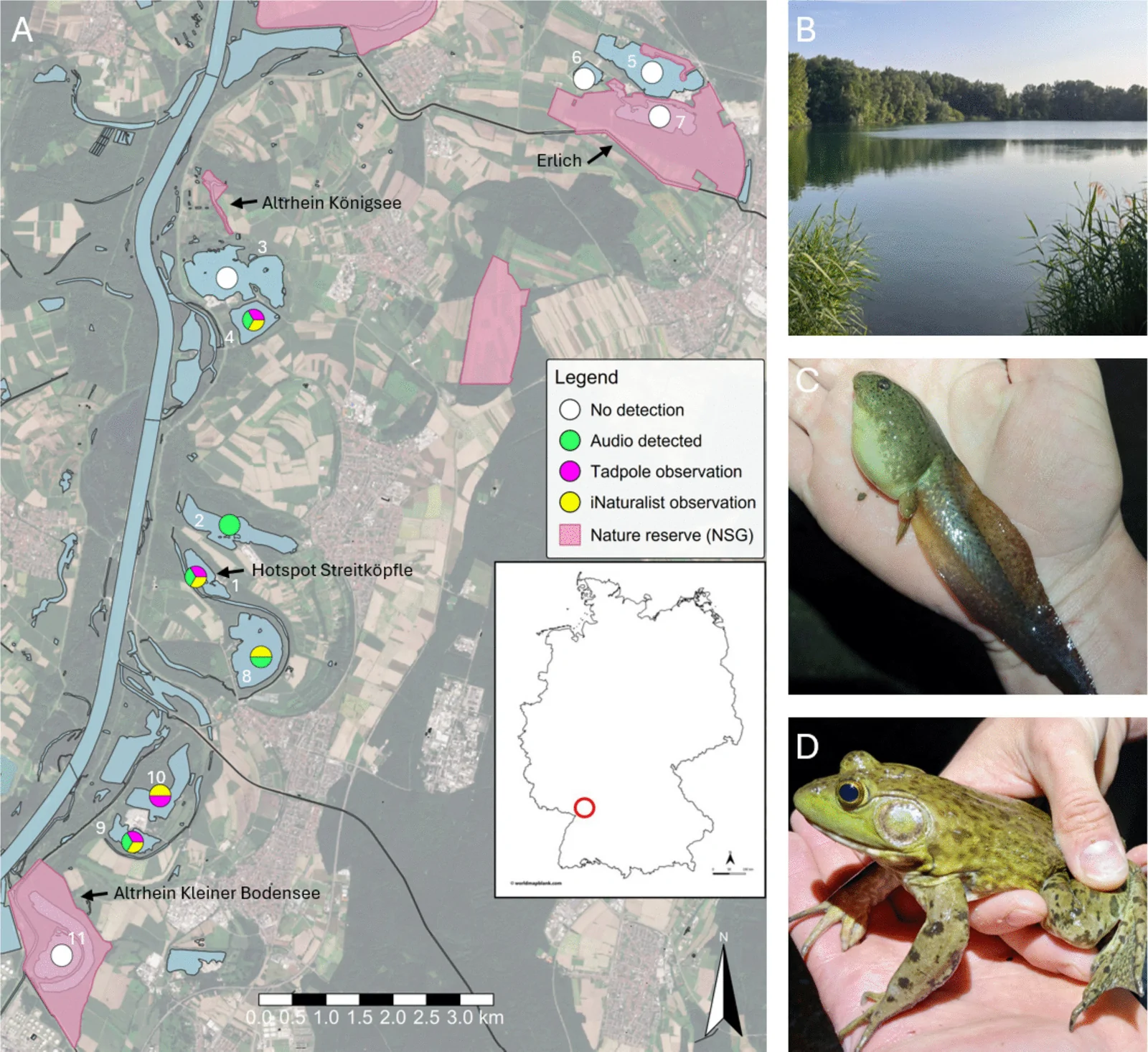
Overview of study sites and the studied species. A Study lakes (light blue, white numbers indicate site ID) in the Upper Rhine region near Karlsruhe, Germany (red circle in inset map), showing bullfrog detections recorded by different survey methods (coloured circles), and nature reserves (light pink) **B** Panoramic view of Lake Streitköpflesee. **C** Tadpole and **D** adult American bullfrog (by Lennhard Hudel, CC-BY, iNaturalist observation ID C = 242549731; D = 216431325)

The literature synthesis identified several key considerations for the application of PAM to invasive species monitoring, including the acoustic detectability of the target species, the monitoring objective and required spatial coverage, and the reliability and validation of automated species detection (Table 1). Here, we use a field-based case study of the invasive American bullfrog (*L. catesbeianus*) to illustrate how these considerations apply in a specific monitoring context. Bullfrogs are particularly suitable for acoustic monitoring because males produce loud and distinctive advertisement calls during the breeding season, while the detection of populations across spatially separated water bodies represents a relevant monitoring task for invasive-species management. We monitored bullfrog calling activity with automated acoustic recorders (AudioMoths firmware version 1.11.0, Hill et al., 2018) at eleven water bodies in the Upper Rhine region near Karlsruhe in southwestern Germany, close to one major hotspot Lake Streitköpflesee in Linkenheim-Hochstetten (49.1196° N, 8.3806° E; Fig. 2A + B). Simultaneous recordings over a 16-day period, from 13 June, 2025 to 29 June, 2025, coinciding with the peak vocal activity and breeding season of bullfrogs in the Upper Rhine region, allowed us to minimize the effects of weather fluctuations, especially temperature changes. During the recording period, weather conditions were consistently warm, with a mean daily air temperature (20 cm above ground) of 24 °C and recorded temperatures ranging from 11 to 36 °C at the Stutensee weather station (Landwirtschaftliches Technologiezentrum Augustenberg, n.d.).

A total of 20 AudioMoths were deployed with a minimum distance of 200 m, distributed between the study locations depending on accessibility (Fig. 2A). All recorders were placed less than five meters away from the shoreline where vegetation varied among lakes. Around gravel lakes, vegetation was often sparse with gravel-based shorelines, while at other lakes, trees and bushes were more dominant with grassy shorelines. Each AudioMoth was programmed to record for 55 s every minute, followed by a 5 s break, from 2 h before sunset until 3 h after sunset at a sampling frequency of 48 kHz. In addition to the AudioMoth recordings, we conducted visual shoreline surveys for bullfrog tadpoles wherever possible. We further compiled all available iNaturalist records of bullfrogs in the study region to assess whether passive acoustic monitoring detected bullfrog presence at sites without previously available public occurrence records.

Maps were created using R version 4.4.0 (R Core Team, 2024) with packages tmap (Tennekes, 2018), terra (Hijmans, 2025), tmaptools (Tennekes, 2025), osmdata (Padgham et al., 2017), sf (Pebesma, 2018; Pebesma & Bivand, 2023), rnaturalearth (Massicotte & South, 2023). The background data for the world map was derived from Sentinel-2 via BKG WMS and OpenStreetMap.

**Fig. 3:**
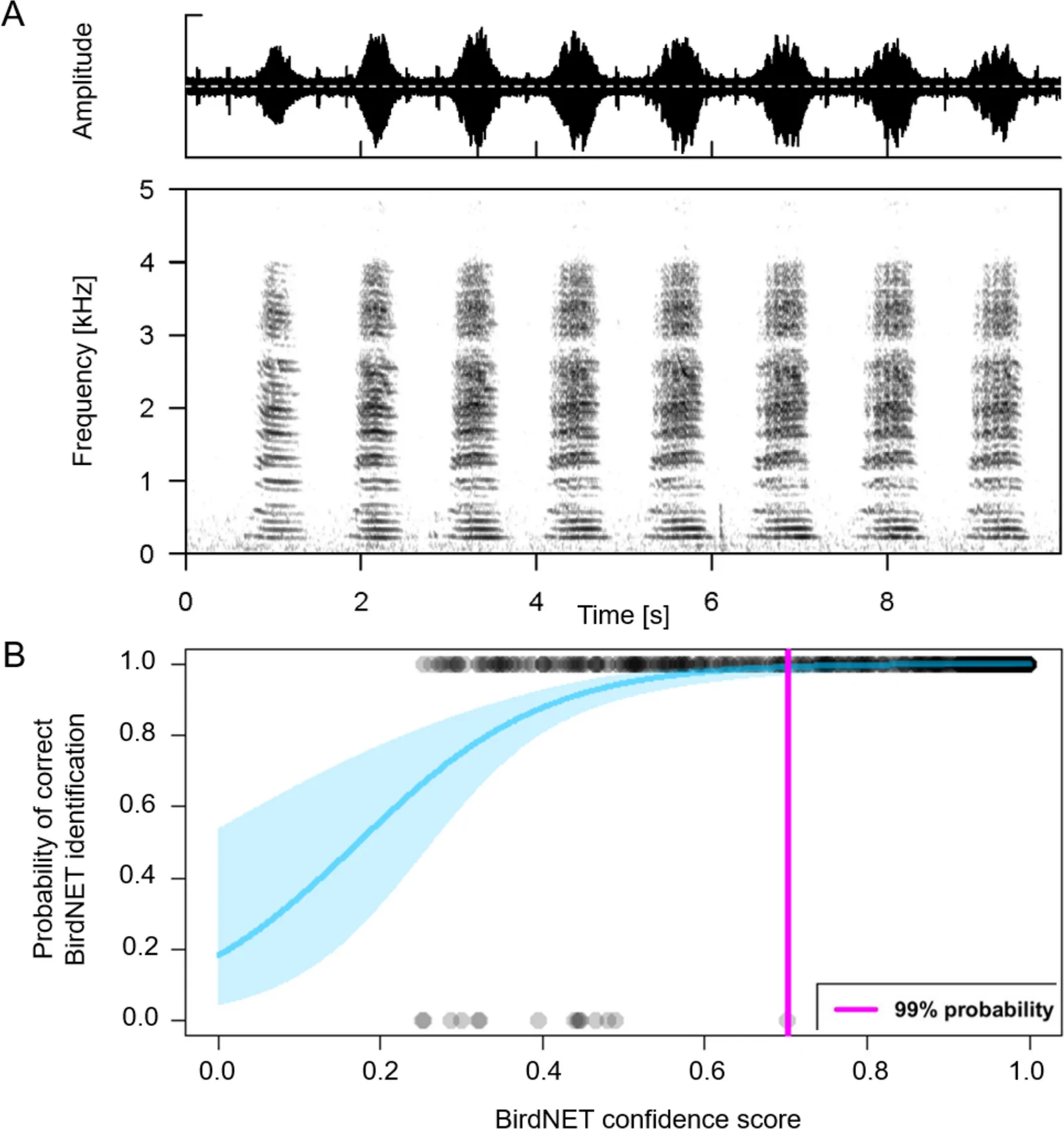
**A** Acoustic characteristics of an American bullfrog (*L. catesbeianus*). The amplitude plot shows changes in call intensity over time with repeated calls lasting about 1 s. The sonogram, depicting frequency (y-axis) over time (x-axis), shows the low-frequency, tonal structure of the calls. **B** Logistic regression showing probabilistic BirdNET score thresholds. The y-axis indicates the probability that the BirdNET prediction is correct (ranging between 0 and 1) at a certain confidence score (x-axis). Grey dots represent correct (1) and incorrect (0) BirdNET identifications. A logistic regression (blue) with 95% confidence intervals (shaded blue areas) was used to determine the BirdNET confidence score thresholds at which the probability of a correct identification was 99%

To automatically detect American bullfrogs in our audio recordings, we used the open-source software BirdNET Analyzer (model version V2.4, sensitivity setting of 1, minimum confidence of 0.25, and an overlap of 2 s; Kahl et al., 2021). BirdNET uses a convolutional neural network which is trained to identify primarily bird songs, however, also a variety of different non-bird species, including dogs, crickets, and American bullfrogs, is covered. Following Wood and Kahl (2024), we validated our bullfrog identifications by randomly extracting 500 audio segments of 5 s containing bullfrog detections, covering the whole range of confidence scores (0.25–1.00) with the implemented function *segments*. We then evaluated whether BirdNET's detections of American bullfrogs were correct (true positive; marked as 1) or incorrect (false positive; marked as 0) by revising the segments’ spectrograms and listening to the audio files in Kaleidoscope version 5.3.8 (Fig. 3A). We used a logistic regression model to determine the BirdNET confidence score thresholds at which the probability of a correct identification was 99% (Fig. 3B). To assess the reliability of this confidence score threshold, we also calculated 95% confidence intervals around this threshold.

**Fig. 4:**
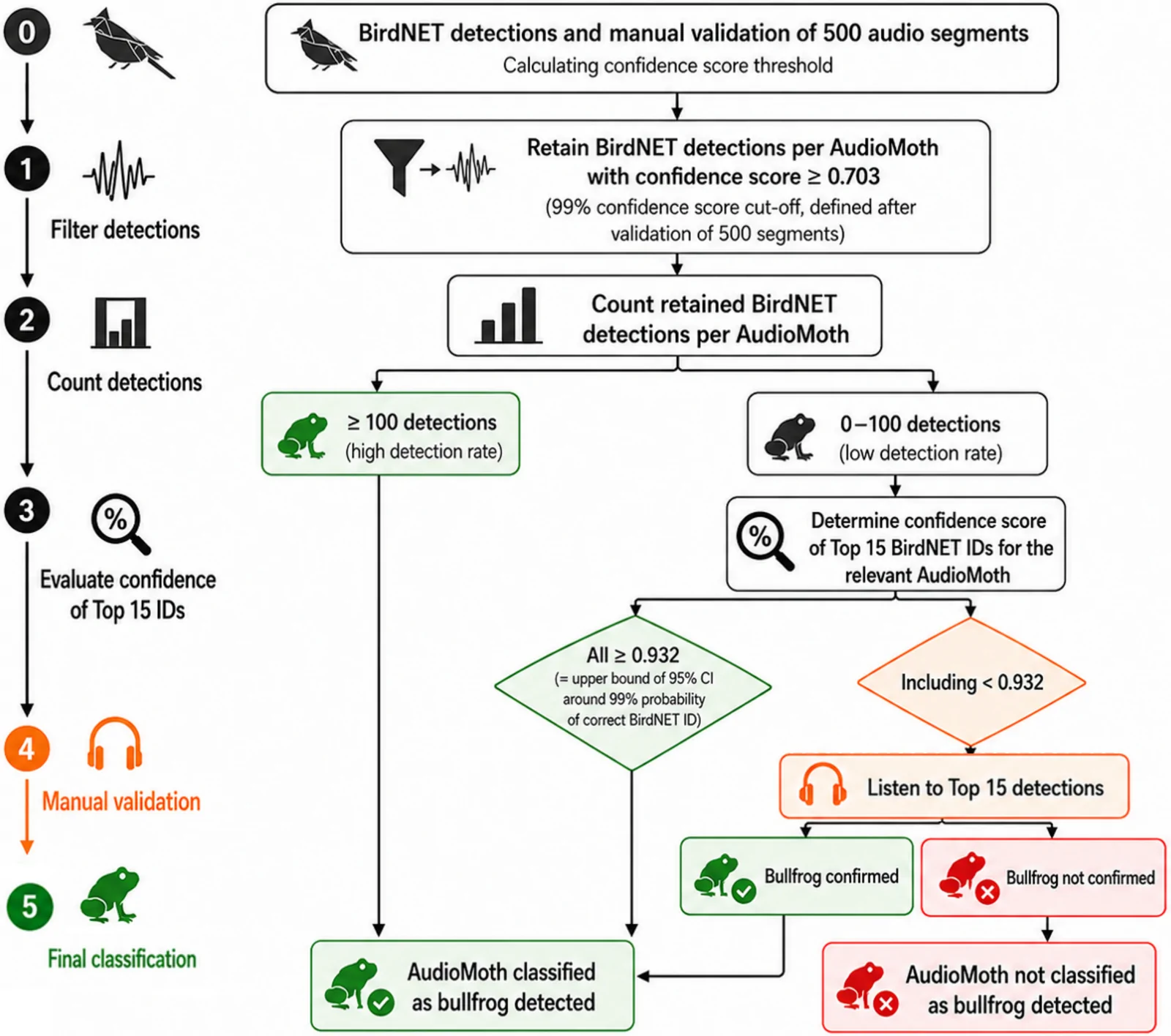
Multi-step process to determine whether an AudioMoth detected bullfrog presence or not, including filtering and counting of raw BirdNET detections above the determined confidence score threshold. For low detection rates, listening to and verifying the top 15 detections might be required. For further information, see text and Supplementary Table S2

To determine if an AudioMoth was classified as “bullfrog detected”, we applied a multi-step decision process based on BirdNET detections and the confidence score thresholds (Fig. 4). First, we counted the number of BirdNET detections above the confidence score threshold (Fig. 4 Step 1). If more than 100 detections were recorded, we directly classified the AudioMoth as “bullfrog detected”. For less than 100 detections, we determined the range of confidence scores of the Top 15 BirdNET detections per AudioMoth (Step 2). In case that all Top 15 detections were above the upper limit of the 95% confidence interval, we could be very sure that the BirdNET detections were actually calls of American bullfrogs (the chance that all 15 of such high confidence scores are actually wrong is very small) and thus directly classify the AudioMoth as “bullfrog detected” (Fig. 4 Step 2). If the Top 15 BirdNET detections included confidence scores within the 95% confidence interval, we manually verified these Top 15 detections by listening to the audio (Fig. 4 Step 3). If a bullfrog call could be confirmed, we classified the AudioMoth as “bullfrog detected”; otherwise, we could not reliably classify the AudioMoth as “bullfrog detected” (Fig. 4 Step 4).

### Results

In total, we recorded 1,600 h (equivalent to over two months of non-stop recordings) of audio recordings which we analyzed with BirdNET Analyzer V2.4 within 24 h. Across these recordings, BirdNET detected 56,298 snippets of 3 s containing bullfrog calls. Out of 500 randomly selected audio segments for subsequent validation, we classified 485 as correctly and 15 as incorrectly identified. Based on this validation, the logistic regression model indicates that BirdNET identifications of American bullfrogs with a confidence score above 0.703 had a 99% probability of being correctly identified (95% confidence interval: confidence score 0.602—0.932; Fig. 3). Using the multi-step verification process (Fig. 4), we could classify 10 out of 20 AudioMoths as “bullfrog detected”, confirming bullfrog invasion for five out of eleven monitored lakes (Supplementary Table S2). Additionally, we observed bullfrog tadpoles at five of these sites, which were also the only sites with available bullfrog occurrence records from citizen science in our study region (Fig. 2; Supplementary Table S2).

## Discussion

### Increasing use of passive acoustic monitoring for invasive animal species

Our systematic review of the scientific literature shows that the use of passive acoustic monitoring for detecting invasive animal species is a relatively recent development which has grown rapidly over the past 14 years. The earliest studies in our dataset were published in 2012 (Buscaino et al., 2012; Kottege et al., 2012), and during the first half of the 2010 s only a small number of publications explicitly addressed monitoring of invasive animal species using acoustic approaches (Kottege et al., 2012, 2015; Tennessen et al., 2013). These early studies were largely exploratory, focusing on proof-of-concept detection methods and characterization of species-specific sounds.

Starting around 2017, both the number of studies and their methodological complexity increased. In amphibians, long-term monitoring and invasion-front studies were conducted for cane toads (Taylor et al., 2017), followed by the development of automated early warning systems (Roe et al., 2018) and large-scale citizen-science initiatives (Rowley et al., 2019). In birds, a series of studies on the invasive barred owl in North America progressively advanced from distribution modelling to near real-time acoustic monitoring integrated with management actions (Jenkins et al., 2025; Rugg et al., 2023; Wood et al., 2020a, 2020b, 2024). Similar methodological shifts are evident in fish, where early automated classifiers for tilapia (Kottege et al., 2012, 2015) were followed by more recent signal discrimination frameworks for invasive weakfish in Europe (Amorim et al., 2023). The most recent studies increasingly employ deep-learning models and large, standardized datasets to enhance transferability and scalability (Bota et al., 2024; Kimura et al., 2025; Leung et al., 2025). At the same time, passive acoustic monitoring has been combined with occupancy modelling and habitat analyses to quantify the environmental factors influencing the occurrence of invasive species (Huck et al., 2024; Ribeiro et al., 2022). Emerging applications extend to mammals (McEwen et al., 2024) and even soil organisms (Keen et al., 2022), indicating a broadening taxonomic scope.

Although the total number of studies remains modest compared to more established biodiversity monitoring approaches, the clear increase of publications within the last five to ten years across multiple taxonomic groups suggests accelerating research interest and increasing methodological maturity. The field appears to be transitioning from isolated, taxon-specific case studies towards more scalable, integrative monitoring frameworks that combine automated detection, ecological modelling, and in some cases direct management intervention (Jenkins et al., 2025; Wood et al., 2024). Taken together, these developments indicate that acoustic monitoring is becoming an increasingly recognized tool in invasion ecology and biosecurity, not only for documenting presence but also for supporting early detection, tracking range expansion, and informing management responses across diverse ecosystems. However, the suitability and effective application of PAM depend strongly on the acoustic ecology of the target species, the monitoring objective and invasion stage, the required spatial scale, and the reliability and validation of acoustic detections. PAM is particularly well suited to taxa that produce frequent, distinctive, and predictable signals, such as birds and amphibians, for which vocalizations play a central role in reproduction and territorial behaviour. Amphibian advertisement calls are largely innate, species-specific, and relatively stereotyped, whereas the songs of many passerine birds are learned and may exhibit pronounced geographic dialects (Köhler et al., 2017; Podos & Warren, 2007). Consequently, bird-sound recognition models may require region-specific training or validation, whereas amphibian-call classifiers may be more transferable across geographic regions. PAM is also highly effective for echolocating bats and cetaceans. In contrast, many terrestrial mammals vocalize too infrequently or unpredictably for PAM to serve as a stand-alone monitoring method, making camera trapping or other complementary approaches more suitable. Nevertheless, PAM can be effective for seasonally or regularly vocal species, including deer during the rut, wolves, and some primates (Enari et al., 2019; Garland et al., 2020). Among arthropods, PAM is especially promising for orthopterans such as grasshoppers and crickets, which produce conspicuous, species-specific stridulatory signals that can be detected and classified automatically across broad temporal and spatial scales (Bennett et al., 2025). For weakly vocal or non-vocal taxa, however, PAM is unlikely to provide comprehensive monitoring on its own and should therefore be combined with complementary approaches such as camera trapping, observer-based surveys, or environmental DNA detection (Hoefer et al., 2026; Larson et al., 2020).

### Passive acoustic monitoring of invasive bullfrogs

Our case study demonstrates the application of several of these considerations in a specific invasive-species monitoring context. Invasive American bullfrogs (*L. catesbeianus*) can be reliably monitored using low-cost passive acoustic monitoring and post-processing under field conditions in Germany. Automated pattern detection with BirdNET, combined with structured manual validation, resulted in species identifications with a 99% probability of being correct (Wood & Kahl, 2024), ensuring that only highly reliable detections were considered. Subsequently, we confirmed bullfrog presence in five of the eleven monitored lakes. However, the absence of confirmed calls at the remaining lakes should not be interpreted as evidence that bullfrogs were absent. Two monitored sites with confirmed bullfrog presence were located within 1 km of the nature reserves “Altrhein Kleiner Bodensee” (No. 2.081) and “Altrhein-Königsee” (No. 2.016), both of which have a protection status broadly comparable to IUCN Category IV. This spatial proximity highlights the importance of continued monitoring in and around these protected areas. Given the high reproductive output and broad predatory diet of American bullfrogs (Adams & Pearl, 2007; Kraus, 2015), the early identification of newly colonized water bodies is ecologically significant, as it allows timely assessment of invasion risk and interventions.

Methodologically, the study shows that existing acoustic classification tools can be reliably applied to invasive amphibian monitoring when combined with structured validation. The successful use of low-cost AudioMoth recorders further shows that reliable detection is not dependent on large-scale acoustic observatories or cloud-based infrastructures (Leung et al., 2025; Roe et al., 2018). Instead, regionally deployable devices combined with clearly defined confidence thresholds and manual verification can produce distribution data of sufficient certainty for applied ecological interpretation.

### Management relevance and local implementation

Invasive species management is often constrained by limited financial and personnel resources, restricted site access, and the need for repeated surveys (Dalton et al., 2024; Haubrock et al., 2021). Passive acoustic monitoring can help address some of these limitations by enabling standardized, repeated data collection with comparatively low field effort, particularly at night when invasive amphibians are most active (Dorcas et al., 2009). For management, its main value lies in confirming persistence at known sites, identifying likely breeding activity through repeated detections of mating calls, and extending monitoring to sites that are difficult to access (Gerhardt & Huber, 2002; Ross et al., 2023). Rather than replacing established methods, acoustic monitoring is best used alongside visual surveys, targeted field checks, and citizen science, where it can help prioritize follow-up actions and support more efficient allocation of limited resources (Hoefer et al., 2026; Sugai et al., 2019).

### Transferability to other invasive species and contexts

Globally, many invasive taxa produce frequent and distinctive sounds, including other amphibians, birds, reptiles and even mammals as shown in our literature review, making them suitable candidates for acoustic monitoring (Bota et al., 2024; Jenkins et al., 2025; Leung et al., 2025; McEwen et al., 2024; Roe et al., 2018; Sugai et al., 2019; Taylor et al., 2017; Wood et al., 2024). More generally, passive acoustic monitoring is particularly useful for species that produce identifiable sounds consistently enough to allow reliable detection over time and across diverse habitats (Ross et al., 2023; Sugai et al., 2019). Importantly, the studies included in our review are likely to represent only a fraction of the potential applications of passive acoustic monitoring of invasive species. Beyond the taxa currently investigated, growing interest exists in applying acoustic methods to flying insects through their flight sounds (Fengler et al., 2026). Wingbeat frequencies have been described as species-specific “acoustic fingerprints” for some invertebrates, suggesting that passive acoustic monitoring could even support detection of invasive insect species in the future (Hall et al., 2025; Kawakita & Ichikawa, 2019; Mankin et al., 2011). For example, acoustic monitoring systems deployed near honeybee hives could enable early detection of the invasive Asian hornet (*Vespa velutina*) as shown by Hall et al. (2025). Coupled with emerging real-time acoustic monitoring technologies, such approaches may provide opportunities for continuous monitoring and rapid management responses to the presence of invasive species (Wood et al., 2024). Commercial systems such as ecoPi (OekoFor GbR, Freiburg, Germany) or BirdWeather PUC (Scribe Labs Inc., California, USA), as well as custom Raspberry Pi-based setups (Raspberry Pi Ltd., Cambridge, UK), can process audio in the field, detect target species automatically, and transmit alerts via cellular networks. Although still a recent and sparsely documented area, these methods have strong potential to enable earlier interventions in invasion ecology.

## Conclusion

Our systematic literature review and case study together demonstrate that acoustic monitoring of animal species is becoming an increasingly useful tool in invasion ecology. Its value lies not only in repeated and cost-effective monitoring over broad temporal and spatial scales, but also in its compatibility with ongoing advances in automated species identification, in-field data processing, and emerging real-time monitoring workflows. When implemented with transparent validation, clear monitoring protocols, and integration with existing survey methods, passive acoustic monitoring has strong potential to support early intervention and to improve the allocation of limited management resources.

## Supplementary Information

Below is the link to the electronic supplementary material.

Supplementary file1 (XLSX 17 KB)

Supplementary file2 (XLSX 47 KB)

## Data Availability

Data availability statement Data are available in the supplementary tables. Furthermore, we uploaded the best recordings for each AudioMoth classified as “bullfrog detected” on www.xeno-canto.org (XC1161554 – XC1161563). See supplementary table S2 for the corresponding ID numbers.

## References

[CR1] Adams, M. J., & Pearl, C. A. (2007). Problems and opportunities managing invasive bullfrogs: Is there any hope? *Biological invaders in inland waters: Profiles, distribution, and threats* (pp. 679–693). Springer Netherlands. 10.1007/978-1-4020-6029-8_38

[CR2] Adriaens, T., Devisscher, S., & Louette, G. (2013). Risk analysis of American bullfrog (*Lithobates catesbeianus*). Risk analysis report of non-native organisms in Belgium. *INBO Report* INBO.R.2013.41. Institute for Nature and Forest Research (INBO), Brussels. 56p.

[CR3] Amorim, M. C. P., Wanjala, J. A., Vieira, M., Bolgan, M., Connaughton, M. A., Pereira, B. P., Fonseca, P. J., & Ribeiro, F. (2023). Detection of invasive fish species with passive acoustics: Discriminating between native and non-indigenous sciaenids. *Marine Environmental Research*. 10.1016/j.marenvres.2023.106017 37178663

[CR4] Bellard, C., Cassey, P., & Blackburn, T. M. (2016). Alien species as a driver of recent extinctions. *Biology Letters,**12*, 20150623. 10.1098/rsbl.2015.0623 26888913 PMC4780541

[CR5] Bennett, D., Nissen, H., Maschke, M. A., Reck, H., & Diekötter, T. (2025). Recent technological developments allow for passive acoustic monitoring of *Orthoptera* (grasshoppers and crickets) in research and conservation across a broad range of temporal and spatial scales. *Basic and Applied Ecology,**84*, 147–157. 10.1016/j.baae.2025.03.004

[CR6] Bisconti, R., Martino, G., Chiocchio, A., Siclari, A., & Canestrelli, D. (2019). Balkan marsh frogs *Pelophylax kurtmuelleri* (Gayda, 1940) introduced in the Aspromonte National Park, southern Italy. *BioInvasions Records,**8*, 26–33. 10.3391/bir.2019.8.1.03

[CR7] Blackburn, T. M., Bellard, C., & Ricciardi, A. (2019). Alien versus native species as drivers of recent extinctions. *Frontiers in Ecology and the Environment,**17*, 203–207. 10.1002/fee.2020

[CR8] Bota, G., Manzano-Rubio, R., Fanlo, H., Franch, N., Brotons, L., Villero, D., Devisscher, S., Pavesi, A., Cavaletti, E., & Pérez-Granados, C. (2024). Passive acoustic monitoring and automated detection of the American bullfrog. *Biological Invasions,**26*, 1269–1279. 10.1007/s10530-023-03244-8

[CR9] Brodie, S., Yasumiba, K., Towsey, M., Roe, P., & Schwarzkopf, L. (2020). Acoustic monitoring reveals year-round calling by invasive toads in tropical Australia. *Bioacoustics,**30*, 125–141. 10.1080/09524622.2019.1705183

[CR10] Buscaino, G., Filiciotto, F., Buffa, G., Di Stefano, V., Maccarrone, V., Buscaino, C., Mazzola, S., Alonge, G., & D’Angelo, S. (2012). The underwater acoustic activities of the red swamp crayfish *Procambarus clarkii*. *Journal of the Acoustical Society of America,**132*, 1792–1798. 10.1121/1.4742744 22978906

[CR11] Dalton, D., et al. (2024). A framework for monitoring biodiversity in protected areas and other effective area-based conservation measures: concepts, methods and technologies, 1st edition. IUCN, International Union for Conservation of Nature. pp. 281–298. Available from https://portals.iucn.org/library/node/51446. Accessed 7 Mar 2026.

[CR12] Demertzis, K., Iliadis, L., & Anezakis, V. D. (2017). A deep spiking machine-hearing system for the case of invasive fish species. In 2017 IEEE International Conference on INnovations in Intelligent SysTems and Applications (INISTA): 23–28.

[CR13] Dorcas, M. E., Price, S. J., Walls, S. C., & Barichivich, W. J. (2009). Auditory monitoring of anuran populations in Amphibian Ecology and Conservation. Oxford University PressOxford. Available from https://academic.oup.com/book/51015/chapter/422431666. Accessed 7 Mar 2026.

[CR14] Early, R., et al. (2016). Global threats from invasive alien species in the twenty-first century and national response capacities. *Nature Communications,**7*, 12485. 10.1038/ncomms12485 PMC4996970 27549569

[CR15] Enari, H., Enari, H. S., Okuda, K., Maruyama, T., & Okuda, K. N. (2019). An evaluation of the efficiency of passive acoustic monitoring in detecting deer and primates in comparison with camera traps. *Ecological Indicators,**98*, 753–762. 10.1016/j.ecolind.2018.11.062

[CR16] Fengler, B., Seyidbayli, C., Reinhardt, A., Anders, M., & Westphal, C. (2026). A review of acoustic systems for insect monitoring: From recording technologies to species recognition performance. *Ecological Informatics,**95*, Article 103733. 10.1016/j.ecoinf.2026.103733

[CR17] Garland, L., Crosby, A., Hedley, R., Boutin, S., & Bayne, E. (2020). Acoustic vs. photographic monitoring of gray wolves (*Canis lupus*): A methodological comparison of two passive monitoring techniques. *Canadian Journal of Zoology,**98*(3), 219–228. 10.1139/cjz-2019-0081

[CR18] Gerhardt, H. C. (1994). The evolution of vocalization in frogs and toads. *Annual Review of Ecology and Systematics,**25*, 293–324.

[CR19] Gerhardt, H. C., & Huber F. (2002). Acoustic communication in insects and anurans: Common problems and diverse solutions. University of Chicago Press. ISBN 0-226-28832-3.

[CR20] Gibb, R., Browning, E., Glover-Kapfer, P., & Jones, K. E. (2019). Emerging opportunities and challenges for passive acoustics in ecological assessment and monitoring. *Methods in Ecology and Evolution,**10*, 169–185. 10.1111/2041-210X.13101

[CR21] Hall, H., Bencsik, M., Capela, N., Sousa, J. P., & De Graaf, D. C. (2025). Remote and automated detection of Asian hornets (*Vespa velutina nigrithorax*) at an apiary, using spectral features of their hovering flight sounds. *Computers and Electronics in Agriculture,**235*, Article 110307. 10.1016/j.compag.2025.110307

[CR22] Haubrock, P. J., et al. (2021). Economic costs of invasive alien species across Europe. *NeoBiota,**67*, 153–190. 10.3897/neobiota.67.58196

[CR23] Hijmans R (2025). terra: Spatial Data Analysis. R package version 1.8-29, https://CRAN.R-project.org/package=terra

[CR24] Hill, A. P., Prince, P., Piña Covarrubias, E., Doncaster, C. P., Snaddon, J. L., & Rogers, A. (2018). AudioMoth: Evaluation of a smart open acoustic device for monitoring biodiversity and the environment. *Methods in Ecology and Evolution,**9*, 1199–1211. 10.1111/2041-210X.12955

[CR25] Hisyam, M., Hestirianoto, T., & Jaya, I. (2020). Sound characteristic of *Procambarus clarkii*. *IOP Conference Series: Earth and Environmental Science,**429*, Article 012036.

[CR26] Hoefer, S., Allen-Ankins, S., McKnight, D., Nordberg, E., & Schwarzkopf, L. (2026). Passive acoustic monitoring outperforms observer-based methods for Australian frog communities. Ecology and Evolutionary Biology. Available from https://ecoevorxiv.org/repository/view/11696/. Accessed 7 Mar 2026.

[CR27] Hopkins, J. M., Higgie, M., & Hoskin, C. J. (2021). Calling behaviour in the invasive Asian house gecko (*Hemidactylus frenatus*) and implications for early detection. *Wildlife Research,**48*, 152–162. 10.1071/WR20003

[CR28] Huck, M. A., Bateman, H. L., de Albuquerque, F. S., & Lewis, J. S. (2024). Anuran occupancy varies with stream characteristics and flow across Arizona wilderness areas. *Freshwater Biology,**69*, 1654–1671. 10.1111/fwb.14334

[CR29] Hulme, P. E. (2006). Beyond control: Wider implications for the management of biological invasions. *Journal of Applied Ecology,**43*, 835–847. 10.1111/j.1365-2664.2006.01227.x

[CR30] Hulme, P. E. (2009). Trade, transport and trouble: Managing invasive species pathways in an era of globalization. *Journal of Applied Ecology,**46*, 10–18. 10.1111/j.1365-2664.2008.01600.x

[CR31] IUCN (2025). The IUCN Red List of Threatened Species. Version 2025-2. Available from https://www.iucnredlist.org. Accessed 28 July 2026.

[CR32] Jenkins, J. M. A., et al. (2025). Congener conflict: Invasive barred owl landscape use across the range of the Northern Spotted Owl. *Journal of Biogeography*. 10.1111/jbi.70070

[CR33] Kahl, S., Wood, C. M., Eibl, M., & Klinck, H. (2021). BirdNET: A deep learning solution for avian diversity monitoring. *Ecological Informatics,**61*, Article 101236. 10.1016/j.ecoinf.2021.101236

[CR34] Kalko, E. K. V., & Schnitzler, H. U. (1989). The echolocation and hunting behavior of Daubenton’s bat, *Myotis daubentoni*. Behavioral Ecology and Sociobiology. *Behavioral Ecology and Sociobiology,**24*, 225–238. 10.1007/BF00295202

[CR35] Kawakita, S., & Ichikawa, K. (2019). Automated classification of bees and hornet using acoustic analysis of their flight sounds. *Apidologie,**50*, 71–79. 10.1007/s13592-018-0619-6

[CR36] Keen, S. C., Wackett, A. A., Willenbring, J. K., Yoo, K., Jonsson, H., Clow, T., & Klaminder, J. (2022). Non-native species change the tune of tundra soils: Novel access to soundscapes of the Arctic earthworm invasion. *Science of the Total Environment*. 10.1016/j.scitotenv.2022.155976 35618134

[CR37] Kimura, K., Fukuyama, I., & Fukuyama, K. (2025). Deep learning-based detector of invasive alien frogs, *Polypedates leucomystax* and *Rhinella marina*, on an island at invasion front. *Biological Invasions*. 10.1007/s10530-025-03553-0

[CR38] Knight, E. C., Hannah, K. C., Foley, G. J., Scott, C. D., Brigham, R. M., & Bayne, E. (2017). Recommendations for acoustic recognizer performance assessment with application to five common automated signal recognition programs. *Avian Conservation and Ecology,**12*(2), 14. 10.5751/ACE-01114-120214

[CR39] Kottege, N., Jurdak, R., Kroon, F., & Jones, D. (2012). Classification of underwater broadband bio-acoustics using spectro-temporal features. Proceedings of the 7th International Conference on Underwater Networks & Systems 19:1–8. 10.1145/2398936.2398961

[CR40] Kottege, N., Jurdak, R., Kroon, F., & Jones, D. (2015). Automated detection of broadband clicks of freshwater fish using spectrooral features. *Journal of the Acoustical Society of America,**137*, 2502–2511. 10.1121/1.4919298 25994683

[CR41] Köhler, J., Jansen, M., Rodríguez, A., Kok, P. J. R., Toledo, L. F., Emmrich, M., Glaw, F., Haddad, C. F. B., Rödel, M.-O., & Vences, M. (2017). The use of bioacoustics in anuran taxonomy: Theory, terminology, methods and recommendations for best practice. *Zootaxa,**4251*(1), 1–124. 10.11646/zootaxa.4251.1.1 28609991

[CR42] Kraus, F. (2015). Impacts from invasive reptiles and amphibians. *Annual Review of Ecology, Evolution, and Systematics,**46*, 75–97. 10.1146/annurev-ecolsys-112414-054450

[CR43] Landwirtschaftliches Technologiezentrum Augustenberg. (n.d.). Stationskarte. Agrarmeteorologie Baden-Württemberg. Retrieved July 23, 2026, from https://www.wetter-bw.de/Agrarmeteorologie-BW/Wetterdaten/Stationskarte

[CR44] Larson, E. R., Graham, B. M., Achury, R., Coon, J. J., Daniels, M. K., Gambrell, D. K., Jonasen, K. L., King, G. D., LaRacuente, N., Perrin-Stowe, T. I., Reed, E. M., Rice, C. J., Ruzi, S. A., Thairu, M. W., Wilson, J. C., & Suarez, A. V. (2020). From eDNA to citizen science: Emerging tools for the early detection of invasive species. *Frontiers in Ecology and the Environment,**18*(4), 194–202. 10.1002/fee.2162

[CR45] Laufer, H., & Waitzmann, M. (2002). Der Ochsenfrosch Rana catesbeiana am nördlichen Oberrhein (Baden-Württemberg). *Herpetofauna,**24*, 5–14.

[CR46] Laufer, H. & Waitzmann, M. (2020). Rote Liste und kommentiertes Verzeichnis der Amphibien und Reptilien Baden-Württembergs. 4. Fassung. Stand 31.12.2020. – Naturschutz-Praxis Artenschutz 16.

[CR47] Leung, B., Lodge, D. M., Finnoff, D., Shogren, J. F., Lewis, M. A., & Lamberti, G. (2002). An ounce of prevention or a pound of cure: Bioeconomic risk analysis of invasive species. *Proceedings of the Royal Society of London. Series B: Biological Sciences,**269*, 2407–2413. 10.1098/rspb.2002.2179 PMC1691180 12495482

[CR48] Leung, F. K. W., Schwarzkopf, L., & Allen-Ankins, S. (2025). Advancing invasive species monitoring: A free tool for detecting invasive cane toads using continental-scale data. *Ecological Informatics*. 10.1016/j.ecoinf.2025.103172

[CR49] Lodge, D. M., et al. (2016). Risk analysis and bioeconomics of invasive species to inform policy and management. *Annual Review of Environment and Resources,**41*, 453–488. 10.1146/annurev-environ-110615-085532

[CR50] Mankin, R. W., Hagstrum, D. W., Smith, M. T., Roda, A. L., & Kairo, M. T. K. (2011). Perspective and promise: A century of insect acoustic detection and monitoring. *American Entomologist,**57*, 30–44. 10.1093/ae/57.1.30

[CR51] Massicotte, P., & South, A. (2023). rnaturalearth: World Map Data from Natural Earth. R package version 1.0.1. https://CRAN.R-project.org/package=rnaturalearth

[CR52] McEwen, B., Bainbridge-Smith, A., Gutschmidt, S., Green, R., & Atlas, J. (2024). An invasive species model and dataset for bioacoustic monitoring of common brushtail possum. *New Zealand Journal of Ecology*. 10.20417/nzjecol.48.3566

[CR53] Nussbaum, R. A., Brodie, E. D., & Storm, R. M. (1983). *Amphibians and reptiles of the Pacific Northwest*. University Press of Idaho.

[CR54] Padgham, M., Rudis, B., Lovelace, R., & Salmon, M. (2017). Osmdata. *The Journal of Open Source Software*. 10.21105/joss.00305

[CR55] Parsons, S., & Szewczak, J. (2009). Detecting, recording and analysing the vocalisations of bats. Ecological and behavioral methods for the study of bats. 2nd edition 91–111.

[CR56] Pebesma, E. (2018). Simple features for R: Standardized support for spatial vector data. *R Journal,**10*(1), 439–446. 10.32614/RJ-2018-009

[CR57] Pebesma, E., & Bivand, R. (2023). Spatial data science: With applications in R. *Chapman and Hall/CRC*. 10.1201/9780429459016

[CR58] Podos, J., & Warren, P. S. (2007). The evolution of geographic variation in birdsong. In Advances in the study of behavior (Bd. 37, S. 403–458). Elsevier. 10.1016/S0065-3454(07)37009-5

[CR59] Pyšek, P., et al. (2020). Scientists’ warning on invasive alien species. *Biological Reviews,**95*, 1511–1534. 10.1111/brv.12627 32588508 PMC7687187

[CR60] R Core Team. (2024). R: A language and environment for statistical computing (Version 4.4.0, “Puppy Cup”). *R Foundation for Statistical Computing, Vienna, Austria.*

[CR61] Raick, X., Huby, A., Kurchevski, G., Godinho, A. L., & Parmentier, É. (2020). Yellow-eyed piranhas produce louder sounds than red-eyed piranhas in an invasive population of Serrasalmus marginatus. *Journal of Fish Biology,**97*, 1676–1680. 10.1111/jfb.14529 32901922

[CR62] Ribeiro, J. W., Harmon, K., Leite, G. A., de Melo, T. N., LeBien, J., & Campos-Cerqueira, M. (2022). Passive acoustic monitoring as a tool to investigate the spatial distribution of invasive alien species. *Remote Sensing*. 10.3390/rs14184565

[CR63] Riede, K. (1993). Monitoring biodiversity: Analysis of Amazonian rainforest sounds. *Ambio*, *22*(8), 546–548. http://www.jstor.org/stable/4314145

[CR64] Roe, P., Ferroudj, M., Towsey, M., & Schwarzkopf, L. (2018). Catching toad calls in the cloud: Commodity edge computing for flexible analysis of big sound data. 4th International Conference on e-Science (e-Science), Amsterdam, Netherlands, 2018, pp. 67–74, 10.1109/eScience.2018.00022

[CR65] Ross, S.R.P.-J., O’Connell, D. P., Deichmann, J. L., Desjonquères, C., Gasc, A., Phillips, J. N., Sethi, S. S., Wood, C. M., & Burivalova, Z. (2023). Passive acoustic monitoring provides a fresh perspective on fundamental ecological questions. *Functional Ecology,**37*, 959–975. 10.1111/1365-2435.14275

[CR66] Rowley, J. J. L., Callaghan, C. T., Cutajar, T., Portway, C., Potter, K., Mahony, S., Trembath, D. F., Flemons, P., & Woods, A. (2019). FrogID: Citizen scientists provide validated biodiversity data on frogs of Australia. *Herpetological Conservation and Biology, 14*, 155–170. https://www.herpconbio.org/Volume_14/Issue_1/Rowley_etal_2019.pdf

[CR67] Rugg, N. M., Jenkins, J. M. A., & Lesmeister, D. B. (2023). Western screech-owl occupancy in the face of an invasive predator. *Global Ecology and Conservation*. 10.1016/j.gecco.2023.e02753

[CR68] Seebens, H., et al. (2017). No saturation in the accumulation of alien species worldwide. *Nature Communications,**8*, 14435. 10.1038/ncomms14435 PMC5316856 28198420

[CR69] Shonfield, J., & Bayne, E. M. (2017). Autonomous recording units in avian ecological research: Current use and future applications. *Avian Conservation and Ecology,**12*(1), 14. 10.5751/ACE-00974-120114

[CR70] Simberloff, D., et al. (2013). Impacts of biological invasions: What’s what and the way forward. *Trends in Ecology & Evolution,**28*, 58–66. 10.1016/j.tree.2012.07.013 22889499

[CR71] Sugai, L. S. M., Silva, T. S. F., Ribeiro, J. W., & Llusia, D. (2019). Terrestrial passive acoustic monitoring: Review and perspectives. *BioScience,**69*, 15–25. 10.1093/biosci/biy147

[CR72] Taylor, A., McCallum, H. I., Watson, G., & Grigg, G. C. (2017). Impact of cane toads on a community of Australian native frogs, determined by 10 years of automated identification and logging of calling behaviour. *Journal of Applied Ecology,**54*, 2000–2010. 10.1111/1365-2664.12859

[CR73] Tennekes, M. (2018). Tmap: Thematic maps in R. *Journal of Statistical Software,**84*(6), 1–39. 10.18637/jss.v084.i0630450020

[CR74] Tennekes, M. (2025). tmaptools: Thematic Map Tools. R package version 3.2. https://CRAN.R-project.org/package=tmaptools

[CR75] Tennessen, J., Parks, S. E., Snow, R. W., & Langkilde, T. L. (2013). Impacts of acoustic competition between invasive Cuban treefrogs and native treefrogs in southern Florida. *The Journal of the Acoustical Society of America,**133*(5), 3535. 10.1121/1.4806381

[CR76] Urbina, J., Bredeweg, E. M., Cousins, C., Blaustein, A. R., & Garcia, T. S. (2020). Reproductive characteristics of American bullfrogs (*Lithobates catesbeianus*) in their invasive range of the Pacific Northwest, USA. *Scientific Reports,**10*, Article 16271. 10.1038/s41598-020-73206-w 33004879 PMC7529884

[CR77] Wengerter, S., Kumlehn, C., Wirsing, T., & Martens, A. (2024). Management des Nordamerikanischen Ochsenfrosches (*Lithobates catesbeianus*): Weiterentwicklung und Evaluation von Fallen und Klangattrappen. RANA, 25.

[CR78] Weterings, R., & Vetter, K. C. (2018). Invasive house geckos (*hemidactylus* spp.): Their current, potential and future distribution. *Current Zoology,**64*, 559–573. 10.1093/cz/zox052 30323835 PMC6178795

[CR79] Wood, C. M., Gutiérrez, R. J., Keane, J. J., & Peery, M. Z. (2020a). Early detection of rapid Barred Owl population growth within the range of the California Spotted Owl advises the precautionary principle. *Condor,**122*, 1. 10.1093/condor/duz058

[CR80] Wood, C. M., Klinck, H., Gustafson, M., Keane, J. J., Sawyer, S. C., Gutiérrez, R. J., & Peery, M. Z. (2020b). Using the ecological significance of animal vocalizations to improve inference in acoustic monitoring programs. *Conservation Biology,**35*, 336–345. 10.1111/cobi.13516 32297668

[CR81] Wood, C. M., & Kahl, S. (2024). Guidelines for appropriate use of BirdNET scores and other detector outputs. *Journal of Ornithology,**165*, 777–782. 10.1007/s10336-024-02144-5

[CR82] Wood, C. M., Günther, F., Rex, A., Hofstadter, D. F., Reers, H., Kahl, S., Peery, M. Z., & Klinck, H. (2024). Real-time acoustic monitoring facilitates the proactive management of biological invasions. *Biological Invasions,**26*, 3989–3996. 10.1007/s10530-024-03426-y

